# Accumulation profiles of PrP^Sc^ in hemal nodes of naturally and experimentally scrapie-infected sheep

**DOI:** 10.1186/1746-6148-9-82

**Published:** 2013-04-19

**Authors:** Rohana P Dassanayake, Thomas C Truscott, M Özgür Özyiğit, Dongyue Zhuang, David A Schneider, Katherine I O’Rourke

**Affiliations:** 1 Department of Veterinary Microbiology and Pathology, College of Veterinary Medicine, Washington State University, Pullman, WA, 99164-7040, USA; 2 U.S. Department of Agriculture, Animal Disease Research Unit, Agricultural Research Service, Pullman, WA, 99164-6630, USA; 3 Department of Pathology, Faculty of Veterinary Medicine, Uludağ University, Görükle, Bursa, 16059, Turkey

**Keywords:** Scrapie, Hemal nodes, Epitope mapping, Sheep, Prions, TSE

## Abstract

**Background:**

In classical scrapie, the disease-associated abnormal isoform (PrP^Sc^) of normal prion protein accumulates principally in the nervous system and lymphoid tissues of small ruminants. Lymph nodes traffic leukocytes via lymphatic and blood vasculatures but hemal nodes lack lymphatic vessels and thus traffic leukocytes only via the blood. Although PrP^Sc^ accumulation profiles are well-characterized in ovine lymphoid tissues, there is limited information on such profiles in hemal nodes. Therefore, the objective of this study was to compare the follicular accumulation of PrP^Sc^ within hemal nodes and lymph nodes by prion epitope mapping and western blot studies.

**Results:**

Our studies found that PrP^Sc^ accumulation in 82% of animals’ abdominal hemal nodes when PrP^Sc^ is detected in both mesenteric and retropharyngeal lymph nodes collected from preclinical and clinical, naturally and experimentally (blood transfusion) scrapie-infected sheep representing all three major scrapie-susceptible *Prnp* genotypes. Abdominal hemal nodes and retropharyngeal lymph nodes were then used to analyze immune cell phenotypes and PrP^Sc^ epitope mapping by immunohistochemistry and PrP^Sc^ banding patterns by western blot. Similar patterns of PrP^Sc^ accumulation were detected within the secondary follicles of hemal nodes and retropharyngeal lymph nodes, where cellular labeling was mostly associated with macrophages and follicular dendritic cells. The pattern of PrP^Sc^ accumulation within hemal nodes and retropharyngeal lymph nodes also did not differ with respect to epitope mapping with seven mAbs (N-terminus, n = 4; globular domain, n = 2; C-terminus, n = 1) in all three *Prnp* genotypes. Western blot analysis of hemal node and retropharyngeal lymph node homogenates revealed identical three banding patterns of proteinase K resistant PrP^Sc^.

**Conclusion:**

Despite the anatomical difference in leukocyte trafficking between lymph nodes and hemal nodes, the follicles of hemal nodes appear to process PrP^Sc^ similarly to lymph nodes.

## Background

Prion diseases, or transmissible spongiform encephalopathies (TSEs), are rare fatal neurodegenerative disorders that affect a number of species including sheep and goats (scrapie), cattle (bovine spongiform encephalopathy, BSE), deer, elk and moose (chronic wasting disease, CWD), mink (transmissible mink encephalopathy), and humans (Creutzfeldt–Jakob disease, CJD). A hallmark of TSEs is the accumulation of a relatively protease-resistant isoform (PrP^Sc^) of the host-encoded normal cellular prion protein (PrP^c^) in the central nervous system [1,2]. In classical scrapie, PrP^Sc^ accumulation in the lymphoreticular system precedes PrP^Sc^ accumulation in the central nervous system [3,4] such that preclinical infection can be detected ante-mortem by immunohistochemical analysis (IHC) of lymphoid follicles associated with the nictitating membrane [5,6], tonsil [7] and rectoanal mucosa [8,9]. Detection of PrP^Sc^ in lymphoid tissues very early in the course of disease suggests that prions may disseminate within the body via the blood and lymphatic vascular systems before entering the central nervous system [3,10]. Indeed, the presence of prions in the peripheral blood of sheep with classical scrapie or with experimental BSE has been confirmed through blood transfusion in lambs [11-14] and in lymphoid tissues of goats and sheep with classical scrapie through bioassay in mice [15-17]. Furthermore, presence of prions or PrP^Sc^ in peripheral blood mononuclear cells was also demonstrated by two in vitro assays such as polyanionic ligand-base enzyme linked immunosorbent (ELISA) [18,19] and protein misfolding cyclic amplification assays [20,21].

Hemal nodes are small independent organs found mostly along larger blood vessels in the head, and thoracic and abdominal viscera in various animal species including sheep [22]. Like the spleen, hemal nodes lack both afferent and efferent lymphatic vessels and therefore receive leukocytes only from the blood, and vascular sinuses are filled with blood rather than lymph [23]. The major functions of hemal nodes are blood filtration and erythrophagocytosis [23]. Despite the lack of lymphatic vessels in the spleen, prions can be readily detected in the spleen as early as three months of age in lambs born to scrapie-infected dams [3,10]. Since prions are present within the different blood components and hemal nodes structurally and functionally resemble spleen, it seems likely that the cellular components of hemal nodes are exposed to prions and thus might accumulate PrP^Sc^ and participate as a site of prion replication.

A previous study in cattle and sheep demonstrated that B- and T-lymphocyte distribution within the primary and secondary follicles of hemal nodes was similar to lymph nodes [24]. Although the lymphocyte and macrophage distribution within hemal node follicles is similar to those of lymph node follicles, it is not clear what type of leukocytes within hemal nodes process PrP^Sc^. It was also not clear whether macrophages, follicular dendritic cells (FDCs) and lymphocytes in hemal nodes or lymph nodes process PrP^Sc^ derived from sheep with natural scrapie differently than those derived after experimental blood transfusion. Therefore, the objectives of this study were to compare PrP^Sc^ distribution and antibody reactivity patterns within hemal nodes and lymph nodes (such as retropharyngeal and mesenteric) collected from naturally or experimentally (intravenous blood component transfusion) exposed scrapie-infected sheep and studied by routine scrapie IHC in conjunction with *in situ* immune cell phenotyping, PrP^Sc^ epitope mapping and western blot studies.

## Results

### PrP^Sc^ accumulates within the hemal nodes of scrapie-infected sheep

Lambs became scrapie infected either through natural exposure or by intravenous transfusion of whole blood or blood cell fractions isolated from scrapie-infected sheep [13]. Transmission of scrapie was confirmed by rectal biopsy [13]. Postmortem tissues were collected at both preclinical and clinical time points and routinely processed for scrapie IHC [9]. PrP^Sc^ accumulation in lymphoid tissues was initially assessed using mAb F99/97.6.1 (Table 1). In this study, once the rectal biopsy was positive, both the retropharyngeal lymph nodes and at least one mesenteric lymph node were positive regardless of genotype, type of disease exposure, and clinical status (Table 2). Making a comparison between abdominal hemal nodes and mesenteric lymph nodes, PrP^Sc^ accumulation in hemal nodes was detected in 82% of the animals. PrP^Sc^ accumulation in the hemal nodes was similarly detected in all three major scrapie susceptible *Prnp* genotypes in both scrapie-infected groups (Table 2). Rectal biopsy from blood transfusion recipients are routinely collected at four months post-transfusion. When the majority of animals became positive for PrP^Sc^ accumulation, animals were then euthanized within four to ten months post-transfusion [13]. Therefore, the lack of PrP^Sc^ detection in the hemal nodes of some preclinical experimental animals is most likely due to the early euthanasia (Table 2).

**Table 1:** **mAbs used in hemal nodes and retropharyngeal lymph nodes PrP**^**Sc **^**epitope mapping IHC study**

**mAb clones**	**Species and isotypes**	**Epitope (PrP**^**c **^**residues)**	**Dilution (μg/mL)**	**Source**
12B2	mouse IgG1	93-97	1.25	gift (Dr. Langeveld)
P4	mouse IgG1	93-99	0.33	R-BioPharm
1E4	mouse IgG1	102-105	16.67	Abcam
8G8	mouse IgG2a	99-114	11.11	Cayman
L42	mouse IgG1	148-153	0.006	R-BioPharm
SAF84	mouse IgG2b	166-172	0.67	Cayman
F99/97.6.1	mouse IgG1	221-225	5.0	VMRD

Abbreviations, mAb = monoclonal antibody; PrP^c^ residues = normal cellular prion amino acid residues.

**Table 2:** **Proportion of scrapie-infected sheep**^**1 **^**in which PrP**^**Sc **^**was detected in abdominal hemal nodes**

***Prnp***	**Total sheep**	**Proportion sheep with PrP**^**Sc **^**detected in hemal nodes**				**Row percentage**
		**Natural exposure**		**Experimental exposure**		
		**Preclinical**	**Clinical**	**Preclinical**^**2**^	**Clinical**	
ARQ/ARQ	30	13/14	8/8	3/6	2/2	***87%***
ARQ/VRQ	24	3/4	3/4	13/16	0	***79%***
VRQ/VRQ	28	3/3	8/8	11/17	0	***79%***
**Column percentage**		***90%***	***95%***	***69%***	***100%***	***82%***

^1^PrP^Sc^ detected by IHC in both the retropharyngeal and a mesenteric lymph node of all sheep.

^2^The preclinical time-point for the experimental exposure of VRQ/VRQ group was at 4-10 months after transfusion of weaned lambs.

### Hemal nodes and lymph nodes show similar anti-prion mAbs reactivity patterns

**Figure 1:**
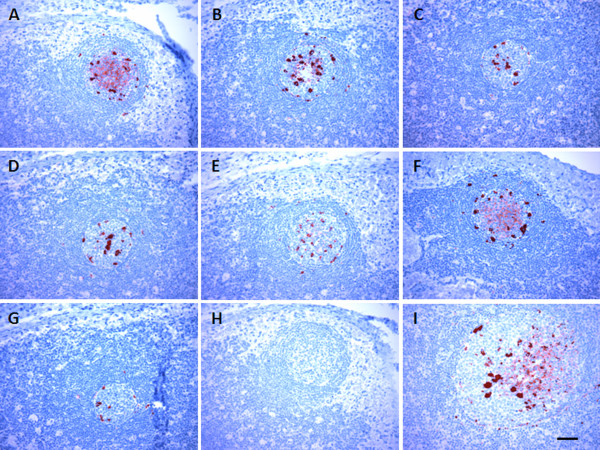
**Immunolabeling of PrP**^**Sc **^**in the hemal nodes from a scrapie-infected sheep.** PrP^Sc^ immunolabeling (dark red) was visible in the secondary follicles of abdominal hemal nodes (animal ID 3777) when labeled with anti-prion mAbs 12B2 (**A**), P4 (**B**), 1E4 (**C**), 8G8 (**D**), L42 (**E**), SAF84 (**F**), F99.97.6.1 (**G**) and retropharyngeal lymph nodes labeled with SAF84 (**I**). The FDC-type of PrP^Sc^ immunolabeling was visible in the hemal nodes of this animal with 12B2 and SAF84 mAbs. PrP^Sc^ immunolabeling was not observed in the hemal nodes’ secondary follicles when anti-prion mAbs were replaced with isotype-matched control immunoglobulins (**H**; same tissue block shown in **A**-**G**). Scale bar = 50 μm.

**Figure 2:**
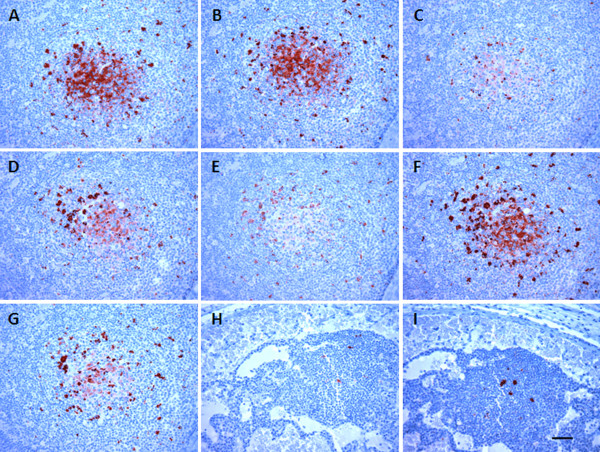
**Immunolabeling of PrP**^**Sc **^**in the retropharyngeal lymph nodes from a scrapie-infected sheep.** The FDC-type of PrP^Sc^ immunolabeling (dark red) was visible in the light zones of secondary follicles in the retropharyngeal lymph nodes (animal ID 3753) when labeled with all seven anti-prion mAbs; 12B2 (**A**), P4 (**B**), 1E4 (**C**), 8G8 (**D**), L42 (**E**), SAF84 (**F**) and F99.97.6.1 (**G**). However, such FDC-type of PrP^Sc^ labeling was not observed in this animal when paired hemal nodes were labeled with any of these antibodies. [Example; F99/97.6.1 (**H**) and SAF84 (**I**)]. Scale bar = 50 μm.

In order to determine whether hemal nodes shows similar PrP^Sc^ distribution and antibody reactivity patterns to lymph nodes, an epitope mapping study of PrP^Sc^ was conducted using a series of mAbs (Table 1) with epitopes representing three major regions of the ovine prion protein: N-terminal (n = 4), globular domain (n = 2) and C-terminal (n = 1). Due to the lack of sufficient amount of mesenteric lymph node blocks from scrapie-infected animals and the routine use of retropharyngeal lymph nodes diagnostic works, paired retropharyngeal lymph nodes and abdominal hemal nodes from naturally and experimentally scrapie-infected sheep, representing all three scrapie susceptible *Prnp* genotypes (Table 3) were used for the epitope mapping study. PrP^Sc^ accumulation within retropharyngeal lymph nodes and abdominal hemal nodes appeared similar (Figures 1 and 2). PrP^Sc^ labeling was detected within the germinal centers of secondary follicles of hemal nodes and retropharyngeal lymph nodes (Figure 1A-G and I). Unlike lymph nodes, dark zone and light zone demarcation within the germinal centers of secondary follicles of the hemal nodes was not very clear. The abundant intracellular pattern of PrP^Sc^ labeling aggregates observed within the germinal centers and mantle zones of hemal nodes are likely to be tangible body macrophages. Hemal nodes collected from several animals in both scrapie-infected groups also showed a wide spread follicular dendritic cell (FDC)-type of PrP^Sc^ labeling pattern which was interspersed between lymphocytes in germinal centers with all seven mAbs. However, the intensity of FDC-type PrP^Sc^ labeling was variable across secondary follicles with different mAbs (Figure 1) and also between different hemal nodes. As it has been previously described [25,26], aggregates of PrP^Sc^ labeling visible in the light zones and dark zones of the germinal centers of secondary follicles and also within the mantle zones and occasionally in paracortical zones of retropharyngeal lymph nodes were macrophages (Figure 2). Unlike in hemal nodes, light zone and dark zone architecture was clearly visible in germinal centers of the secondary follicles in retropharyngeal lymph nodes where well-defined FDC-type PrP^Sc^ labeling was clearly interspersed between lymphocytes in the light zones (Figure 2). In some animals, FDC-type PrP^Sc^ labeling was not very strong with mAbs 1E4 and L42 (Figure 2C and E). The intensities of FDC-type PrP^Sc^ labeling patterns varied between follicles, tissue blocks and animals. However, the majority of naturally and experimentally scrapie-infected animals with different *Prnp* genotypes showed both macrophages and FDC-type PrP^Sc^ labeling. PrP^Sc^ labeling was not detected from three hemal node sections from three naturally scrapie-infected sheep with 1E4 and 8G8 mAbs and three hemal node sections from two experimentally scrapie-infected animals with 1E4 and 12B2 mAbs (Table 3). Labeling of the sections from the same tissue blocks from these sheep with these mAbs failed to identify PrP^Sc^ positive follicles due to sectioning through the PrP^Sc^ positive areas within these nodes. PrP^Sc^ immunolabeling was not observed when hemal nodes and retropharyngeal lymph nodes from scrapie-negative sheep were incubated with anti-prion mAbs or when hemal nodes and retropharyngeal lymph nodes from scrapie-infected animals were incubated with isotype-matched immunoglobulins (Figure 1H).

**Table 3:** **PrP**^**Sc **^**epitope mapping immunolabeling results of hemal nodes and retropharyngeal lymph nodes from naturally and experimentally scrapie-infected sheep**

**Animal ID**	**Age (months)**	**Source of scrapie**	**Clinical status**	***Prnp***	**Anti-prion mAbs**						
					**12B2**	**P4**	**1E4**	**8G8**	**L42**	**SAF84**	**F99/97.6.1**
					**(Hemal nodes/Lymph nodes)**						
											
3339	37	Natural	Preclinical	ARQ/ARQ	/	/	/	/	/	/	/
4071	29	Natural	Preclinical	ARQ/ARQ	/	/	/	/	/	/	/
3403	37	Natural	Clinical	ARQ/ARQ	/	/	n	/	/	/	/
3727	NA	Natural	Clinical	ARQ/ARQ	/	/	/	n	/	/	/
3768	30	Natural	Clinical	ARQ/ARQ	/	/	/	/	/	/	/
4063	29	Natural	Clinical	ARQ/VRQ	/	/	/	/	/	/	/
2933	46	Natural	Clinical	ARQ/VRQ	/	/	/	/	/	/	/
3588	30	Natural	Clinical	ARQ/VRQ	/	/	/	/	/	/	/
3753	35	Natural	Clinical	VRQ/VRQ	/	/	/	/	/	/	/
3755	12	Natural	Clinical	VRQ/VRQ	/	/	n	/	/	/	/
3774	48	Natural	Clinical	VRQ/VRQ	/	/	/	/	/	/	/
3776	50	Natural	Clinical	VRQ/VRQ	/	/	/	/	/	/	/
3777	50	Natural	Clinical	VRQ/VRQ	/	/	/	/	/	/	/
3806	28	Natural	Clinical	VRQ/VRQ	/	/	/	/	/	/	/
3807	25	Natural	Clinical	VRQ/VRQ	/	/	/	/	/	/	/
3823	13	Exp- BC	Preclinical	ARQ/ARQ	/	/	/	/	/	/	/
3820	24	Exp- PRP	Preclinical	ARQ/ARQ	n	/	n	/	/	/	/
3628	12	Exp- WB	Preclinical	ARQ/VRQ	/	/	/	/	/	/	/
4132	13	Exp- WB	Preclinical	ARQ/VRQ	/	/	/	/	/	/	/
4118	13	Exp- PBMC	Preclinical	ARQ/VRQ	/	/	/	/	/	/	/
4494	16	Exp- PBMC	Preclinical	VRQ/VRQ	/	/	/	/	/	/	/
4381	11	Exp- B cells*	Preclinical	VRQ/VRQ	/	/	/	/	/	/	/
4382	11	Exp- B cells*	Preclinical	VRQ/VRQ	/	/	n	/	/	/	/
4385	11	Exp- B cells	Preclinical	VRQ/VRQ	/	/	/	/	/	/	/
4489	16	Exp- B cells	Preclinical	VRQ/VRQ	/	/	/	/	/	/	/

Abbreviations: *Prnp* = prion genotypes; NA = not available; EXP = experimental; PRP = platelet-rich plasma; BC = buffy coat; WB = whole blood; PBMC = peripheral blood mononuclear cells; * = CD21^+^ subset of B cells; blank = PrP^Sc^ labeling was detected; n = PrP^Sc^ labeling was not detected.

### Macrophages and FDCs within the hemal nodes and retropharyngeal lymph nodes are the major cell types to harbor PrP^Sc^

**Figure 3:**
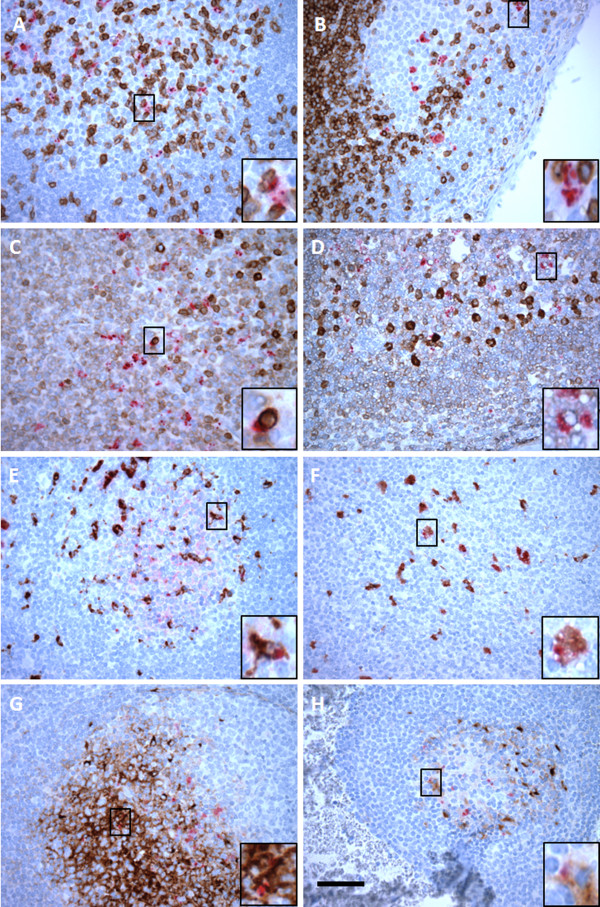
**Double immunolabeling of leukocyte subsets and PrP**^**Sc **^**in the hemal nodes and retropharyngeal lymph nodes from scrapie-infected sheep.** Retropharyngeal lymph nodes (**A, C, E, G**) and abdominal hemal nodes (**B, D, F, H**) were immunolabeled with T lymphocyte-specific (CD3; animal ID 4496; **A** and **B**), B lymphocyte-specific (CD79a; animal ID 4496; **C** and **D**), macrophage-specific (CD163; animal ID 4489; **E** and **F**) and follicular dendritic cell-specific (120 kDa antigen; animal ID 3776; **G** and **H**) Abs along with anti-prion F99/97.6.1 mAb. Under these double labeling conditions, PrP^Sc^ appears in red while each cell-type appears in brown. Enlarged pictures of co-labeled cells with cell-type specific mAb and PrP^Sc^ are inserted into the right lower corner of each panel. Scale bar = 50 μm.

We used leukocyte subset-specific mAbs (Table 4) to phenotype PrP^Sc^ accumulating cells *in situ* and mAb F99/97.6.1 to label PrP^Sc^. T lymphocytes were observed predominantly within the paracortex or interfollicular regions of retropharyngeal lymph nodes but some were also found scattered in the light zone in the germinal centers of secondary follicles (Figure 3A). In hemal nodes, T lymphocytes were found around the follicles, few in the germinal centers (Figure 3B) and also in the interfollicular regions when two follicles were next to each other. B lymphocytes were abundant in the germinal centers and mantle zones of hemal node and retropharyngeal lymph node secondary follicles (Figure 3C and 3D). It has been previously reported that there is occasional accumulation of PrP^Sc^ granules on the outer surface of B and T lymphocytes plasma membranes [27]. Using our method, we also observed similar PrP^Sc^ labeling near the outer surface of B and T lymphocytes in both tissues (Figure 3A-D). PrP^Sc^ labeling was predominantly found within macrophages (Figure 3E and 3F) and FDCs (Figure 3G and 3H) within the germinal centers of both types of nodes. Although FDC-type PrP^Sc^ labeling in the light zones was very prominent in single labeling (Figures 1 and 2), such labeling was significantly reduced under double labeling conditions. Also, it is noteworthy to mention that despite using the same immunolabeling conditions, FDC labeling was very strong with retropharyngeal lymph node follicles compared to hemal node follicles (Figure 3G and H).

**Table 4:** mAbs used to identify leukocyte subsets in hemal and retropharyngeal lymph nodes during immunohistochemistry study

**Cell types**	**Cell surface marker**	**mAb clones**	**Species and isotypes**	**Dilution (μg/mL)**	**Source**
T ymphocytes	CD3	A0452	Rabbit poly	3.0	Dako
B lymphocytes	CD79a	MCA2538	mouse IgG1	50.0	AbD Serotec
FDCs	120 kDa	CAN.42	mouse IgM	4.1	Dako
Mac	CD163	MCA1853	mouse IgG1	33.3	AbD Serotec

Abbreviations, mAb = monoclonal antibody; poly = polyclonal antibody; FDCs = follicular dendritic cells; Mac = macrophages.

### PrP^Sc^ banding patterns in hemal nodes and retropharyngeal lymph nodes are similar

**Figure 4:**
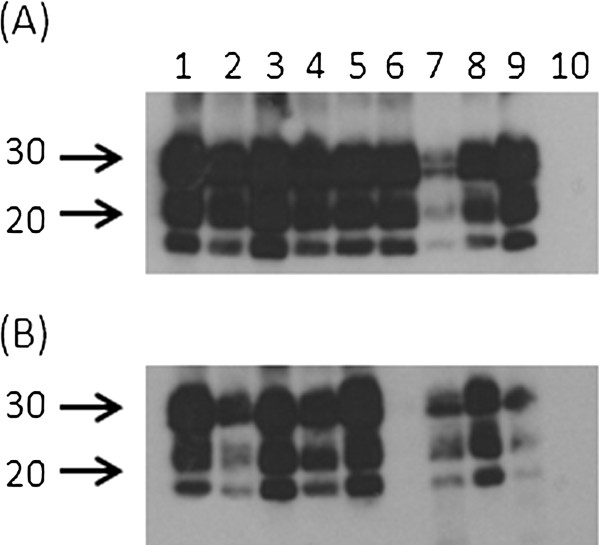
**Detection of characteristic proteinase K resistant three isoforms of PrP**^**Sc **^**from hemal nodes and retropharyngeal lymph nodes by western blot assays.** Western blot analysis was performed with retropharyngeal lymph node (**A**) and abdominal hemal node (**B**) homogenates prepared from experimentally and naturally scrapie-infected sheep. Lanes 1 – 6, experimentally (blood transfusion recipient) scrapie-infected sheep (ID 4489, 4493, 4494, 4495, 4496, and 4498 respectively); lanes 7 -9 naturally scrapie-infected sheep (ID 3349, 3779 and 3781 respectively). Lane 10, scrapie uninfected control sheep (ID 4492). To concentrate PrP^Sc^, proteinase K treated samples were incubated with sodium phosphotungstic acid and PrP^Sc^ was detected using F99/97.6.1 mAb. The positions of the molecular mass markers (in kDa) are shown to the left of the blots.

Epitope mapping using seven different mAbs confirmed that accumulated PrP^Sc^ in hemal nodes and retropharyngeal lymph nodes appears to be similar. However, such IHC studies alone are unable to provide any information regarding PrP glycosylation or molecular mass differences. Thus, PrP^Sc^-specific WB assays were performed with F99/97.6.1 mAb. Phosphotungstic acid (PTA) precipitation was used to concentrate PrP^Sc^ from the samples. Retropharyngeal lymph node homogenates from experimentally scrapie-infected sheep and naturally scrapie-infected sheep had the characteristic three banding pattern of proteinase K resistant PrP^Sc^ (Figure 4A). One animal in the naturally scrapie-infected group had weak PrP^Sc^ signals (Figure 4A, lane 7). Except for one animal from the experimental group, (Figure 4B, lane 6), hemal nodes from experimentally and naturally scrapie-infected sheep also showed the characteristic proteinase K resistant three banding pattern of PrP^Sc^ (Figure 4B). Interestingly, PrP^Sc^ immunolabeling in the hemal nodes of this animal (Figure 4B, lane 6) was clearly detected by IHC with the same F99/97.6.1 mAb (data not shown). Hemal nodes from one animal in the naturally scrapie-infected group had weak PrP^Sc^ signals (Figure 4B, lane 9). As expected, the scrapie-uninfected control sheep did not show any proteinase K-resistant PrP bands (Figure 4A and B, lane 10). The distribution of PrP^Sc^ bands in both types of nodes were similar and they were in the order of diglycosylated > monoglycosylated > unglycosylated. Molecular masses of di- (26.61-27.20 kDa), mono- (19.96-20.77 kDa) and un-glycosylated (16.22-16.77 kDa) PrP^Sc^ bands in hemal nodes and corresponding retropharyngeal lymph nodes from naturally and experimentally scrapie-infected sheep were similar.

## Discussion

The goal of this study was to compare the distribution and accumulation of PrP^Sc^ in hemal nodes and a representative peripheral lymph node such as the retropharyngeal or mesenteric lymph node. In addition, we were interested to know if these characteristics were dependent on *Prnp* genotype or type of exposure. Our findings revealed that PrP^Sc^ labeling was detected in hemal nodes of 82% sheep with PrP^Sc^ positive in both mesenteric and retropharyngeal lymph nodes. The lack of PrP^Sc^ detection in hemal nodes of 15 scrapie-infected sheep included both naturally and experimentally infected, and preclinical as well as clinical sheep (Table 2). Since we only collected and evaluated few hemal nodes from thoracic and abdominal regions, the lack of PrP^Sc^ detection in these sheep does not necessarily mean their hemal nodes are negative for prions. Perhaps other hemal nodes in head region or abdominal viscera could have accumulated PrP^Sc^. We did not observe any differences in PrP^Sc^ epitope mapping between hemal nodes and retropharyngeal lymph nodes of scrapie-infected sheep (Figures 1 and 2) or any difference in lymphocytes, macrophages or FDCs arrangement within the follicles of hemal and lymph nodes (Figure 3). Therefore, these observations suggest that irrespective of mode of infection (natural or blood transfusion) or vascular anatomical differences (blood only, hemal nodes; blood and lymphatics, lymph nodes), once prions enter into follicles, both hemal and lymph nodes process PrP^Sc^ similarly.

After ingestion of scrapie contaminated materials, PrP^Sc^ accumulation has been first detected within the follicles of Peyer’s patches and draining gut-associated (mesenteric) lymph node (GALNs) in the ileum [3]. Dissemination of prions from draining GALNs to most of the non-gut associated secondary lymph nodes is not clear. A recent study in mice by blocking the sphingosine 1-phosphate receptor which sequesters lymphocytes within secondary lymph nodes revealed that B lymphocytes disseminate prions from gut associated lymph nodes to other secondary lymph nodes [28]. Previous studies of cryopreserved hemal and lymph nodes showed that B lymphocytes within the germinal centers of secondary follicles of sheep are positive for CD21 [22,29]. Since CD21-positive B lymphocytes are known to be recirculating B lymphocytes [30,31], it is possible that hemal nodes receive prions mainly through CD21- positive B lymphocytes. Under our IHC labeling conditions, PrP^Sc^ labeling was observed near the outside surface of B lymphocytes (Figure 3C-D). However, based on the high resolution electron microscopic observations of submandibular lymph nodes by McGovern and Jeffrey [25], the observed PrP^Sc^ labeling near the outside surface of B and T lymphocytes are most likely to be PrP^Sc^ in the FDC dendrites. Blood transfusion experiments conducted in lambs [11-14,32,33] and IC inoculation in transgenic Tg338 mice [14,21] revealed that infectious prions are associated with platelets and all major PBMC subsets of scrapie-infected sheep. Our inability to transmit scrapie using only platelet-poor plasma [13] prompted us to speculate PrP^Sc^ was not in a cell-free form in ovine plasma. However, a recent study by Bannach et al., 2012 [34] with fluorescent antibody probe revealed the presence of PrP^Sc^ in plasma samples collected from scrapie-infected sheep whose plasma samples were previously identified as PrP^Sc^ negative by IDEXX ELISA [18]. Since hemal nodes receive blood rather than lymph, it is possible that hemal nodes can also receive a cell-free form of PrP^Sc^ from the blood circulation as well.

Hemal nodes and retropharyngeal lymph nodes were evaluated by WB assays to detect characteristic PrP^Sc^ banding patterns after proteinase K treatment. It has been previously reported that sodium phosphotungstic acid (PTA) can enrich PrP^Sc^ in the samples [35]. Therefore, PrP^Sc^ in homogenates from both types of nodes were concentrated with PTA before WB assays. Hemal node and retropharyngeal lymph node homogenates from naturally and experimentally scrapie-infected sheep showed typical banding patterns of three PrP^Sc^ isoforms (di-, mono-, and un-glycosylated; Figure 4A and B). Although PrP^Sc^ banding pattern was not observed in one animal’s hemal node preparations in WB assay, positive PrP^Sc^ immunolabeling was clearly detected in several other hemal nodes separately collected from the abdominal cavity for IHC. However, such PrP^Sc^ labeling was not detected from thoracic hemal nodes. The lack of PrP^Sc^ accumulation in the hemal nodes collected for WB assay from the abdominal cavity and positive IHC labeling in hemal nodes from abdominal cavity but not from the thoracic cavity in this particular animal further confirmed that PrP^Sc^ accumulation is not uniform among hemal nodes. Therefore, this finding highlights that the lack of PrP^Sc^ accumulation detected in the hemal nodes in some animals (18%) compared to those of mesenteric lymph nodes, is most likely due to the limited number of hemal nodes collected and assessed by IHC. Although no attempt was made to perform glycoform analysis due to insufficient tissue, there were no obvious differences in glycosylation patterns between both types of tissues or between naturally and experimentally scrapie-infected sheep. Furthermore, molecular masses of PrP^Sc^ bands of hemal nodes were similar to corresponding lymph nodes. Therefore, these findings further confirmed our IHC observations that PrP^Sc^ in both types of tissues are fundamentally similar to each other and thus both tissues process PrP^Sc^ similarly.

The IHC analyses of hemal nodes collected from scrapie-infected sheep demonstrated the presence of both primary and secondary follicles. Unlike in retropharyngeal lymph nodes, dark and light zone architecture within the germinal centers of secondary follicles in hemal nodes was not very clear. However, observed FDC-type PrP^Sc^ labeling interspersed between lymphocytes in the germinal centers in some secondary follicles strongly suggest the presence of light zones and dark zones in the hemal nodes as well (Figure 1A and 1F). Since the prion protein is a self-antigen, it is unlikely that germinal center formation within the secondary follicles of hemal and lymph nodes were induced by PrP^Sc^. Whatever the cause for such germinal center formation, these observations suggest that hemal nodes of sheep are not simply passive organs that accumulate plasma or blood-derived PrP^Sc^ but rather immunologically active organs which replicate and process PrP^Sc^ similarly to lymph nodes.

## Conclusions

A PrP^Sc^ epitope mapping study using seven different mAbs by IHC revealed that PrP^Sc^ labeling patterns of scrapie-infected ovine hemal nodes are similar to those of lymph nodes; and macrophages and FDCs are the major cell types to harbor PrP^Sc^ in both types of nodes. Western blot analysis confirmed that glycosylation patterns and molecular masses of PrP^Sc^ in hemal and lymph nodes are similar. Therefore, we concluded that hemal nodes process prions similar to lymphoid tissues.

## Methods

### Naturally and experimentally scrapie-infected sheep

All experimental protocols used in this study were approved by the Institutional Animal Care and Use Committee (IACUC) at Washington State University before onset of the study. The first group of animals (n = 41) included naturally scrapie-infected sheep and they were part of a larger ongoing study. Some of the naturally scrapie-infected animals were born and raised at a persistently scrapie-infected USDA animal research unit at Pullman, WA and developed either preclinical or clinical scrapie. Some of the other naturally scrapie-infected sheep were received from privately owned scrapie-infected flocks quarantined at USDA facilities and subsequently developed clinical scrapie. All of the experimentally scrapie-infected animals (n = 41) used in this study were part of our previous blood transfusion study. Blood fractions transfused, housing, rectal biopsy and necropsy findings were described in our previous publication [13]. Both groups of sheep were mixed breeds of white face, black face sheep of the Polypay, Suffolk or Columbia breeds and the ages of these animals were in the range of one to five years at the time of necropsy. At necropsy, along with the other tissues, an average of four to eight abdominal and thoracic hemal nodes were also collected and initially evaluated for the presence of PrP^Sc^ after immunohistochemistry labeling with anti-PrP mAb F99/97.6.1 [6]. PrP^Sc^ positive abdominal hemal nodes from two preclinical (2 ARQ/ARQ) and thirteen clinical (4 ARQ/ARQ, 2 ARQ/VRQ and 7 VRQ/VRQ) naturally scrapie-infected sheep and 10 preclinical (2 ARQ/ARQ, 3 ARQ/VRQ and 5 VRQ/VRQ) experimentally scrapie-infected sheep were selected for prion epitopes mapping study. The *Prnp* genotypes of all the sheep were determined by sequencing of open reading frame of the *Prnp* gene as described before [36]. Genotypes are shown by the deduced amino acid residues at positions 136, 154 and 171 respectively. Sheep with *Prnp* genotypes encoding alanine (A) or valine (V) at 136, arginine (R) at 154 and glutamine (Q) at 171 were selected for this study. *Prnp* genotypes and scrapie status are shown in Table 3.

### Detection of PrP^Sc^ in hemal nodes by immunohistochemistry

Postmortem abdominal hemal nodes, mesenteric and retropharyngeal lymph nodes were fixed in formalin and processed according to standard procedures to prepare paraffin embedded tissue blocks. Both hemal nodes and retropharyngeal lymph nodes from the same animal were serially sectioned at 2-3 μm thicknesses. At least eight sections were prepared for each type of node for every animal. One serial section each of hemal node and retropharyngeal lymph node from the same animal was placed onto a treated glass slide (Superfrost®/Plus, Fisher Scientific, Pittsburg, PA). Deparaffinization, antigen retrieval and immunolabeling were performed using an automated processor (Benchmark, Ventana Medical Systems, Tucson, AZ). Antigen retrieval was accomplished in two parts. First by a three minute 98% formic acid treatment of the slides followed by Trizma® pH 7.0 (Sigma, St Louise, MO) buffered washes before loading onto the automatic stainer. Then, secondly, by the automatic stainer using proprietary reagent CC1 (Tris buffer) on an standard automatic program including deparaffinization. Each of these slides was separately immunolabeled with one of four N-terminal specific mAbs: 12B2 (a kind gift of J. Langeveld, Central Institute for Animal Disease Control, Lelystad, The Netherlands), P4 (R-BioPharm Inc., Washington, MO), 1E4 (Abcam Inc., Cambridge, MA), 8G8 (Cayman Chemical Company, Ann Arbor, MI); two globular domain specific: L42 (R-BioPharm), SAF84 (Cayman); and one C-terminal specific: F99/97.6.1 (VMRD Inc., Pullman, WA) anti-prion mAbs. After the primary Abs, slides were incubated with biotinylated secondary Abs and detected with a streptavidin-horseradish peroxidase (HRP) conjugate and 3-amino-9-ethylcarbazole as the dark red chromagen using the Basic AEC Detection Kit (Ventana). Slides were counterstained with hematoxylin. The list of anti-prion mAbs with their corresponding epitope recognition residues and concentrations are shown in Table 1. Hemal nodes were considered positive for prion when PrP^Sc^ accumulation was detected in at least in one follicle. Retropharyngeal lymph nodes collected from scrapie positive and negative animals were used in each run as control samples. Samples were considered positive for PrP^Sc^ if coarse dark-red deposits were detected in the follicles by bright-field microscopy. Immunolabeling intensities of the positive control samples were equivalent for all runs. Photomicrographs were taken with an Olympus BX40 microscope coupled with an Olympus Q-Color3 camera. Axiovision software was used for scaling and Adobe Photoshop Elements 5.0 for formatting.

### Identification of PrP^Sc^ positive leukocytes in hemal nodes by immunohistochemistry

Double immunolabeling of hemal nodes and retropharyngeal lymph nodes was performed with cell-surface specific marker mAbs for B- and T-lymphocytes, follicular dendritic cells or macrophages and the anti-prion specific mAb F99/97.6.1. The list of mAbs and polyclonal Abs with their leukocyte subset specificity and concentration used are indicated in Table 4. HRP-conjugated horse anti-mouse IgG (Vector Laboratory Inc., Burlingame, CA), Ventana universal secondary Ab and HRP-conjugated rat anti-mouse IgM (SouthernBiotech, Birmingham, AL) were used as secondary antibody. The double-immunolabeling procedure was also performed using an automated processor (Discovery XT, Ventana Medical Systems). Briefly, hemal nodes and retropharyngeal lymph node sections on the same slide were first incubated with cell-specific Abs followed by incubation with the appropriate HRP-conjugated secondary Abs and detection with 3, 3’-diaminobenzadine as the chromagen using Discovery® DAB Map Kit (Ventana). These slides were again incubated with anti-prion mAb F99/97.6.1 followed by incubation with Ventana universal secondary biotinylated secondary Ab and streptavidin conjugated alkaline phosphatase and detected with Fast Red/Naphthol chromogen using Discovery® RedMap Kit (Ventana). All the slides were counterstained with hematoxylin. Under these conditions, labeled leukocytes appeared in brown while PrP^Sc^ appeared in bright red. Photomicrographs were taken and processed as described in the previous section.

### PTA-western blot assay

Due to the lack of preserved frozen nodes from naturally scrapie-infected sheep which were used in the epitope mapping study, paired hemal and retropharyngeal lymph nodes collected from three other naturally scrapie-infected sheep (ARQ/ARQ *Prnp* genotype) were used for WB assays. In addition to two experimentally scrapie-infected sheep (4489 and 4494) used in the epitope mapping study, paired hemal and retropharyngeal lymph nodes collected from four other blood transfusion recipients (VRQ/VRQ *Prnp* genotype) were also included in WB assays. To concentrate PrP^Sc^ from samples, sodium phosphotungstic acid (PTA) was used as described previously [35,37]. Two to four hemal nodes (50 – 100 mg) or retropharyngeal lymph nodes (150 mg) from each animal were minced, resuspended in 1 mL lysis buffer and homogenized. Then the homogenates were treated with collagenase (2.5 mg/mL at 37°C for overnight). Samples were treated with sarkosyl and DNAse I and supernatants were collected after centrifugation. Incubation of these supernatants with proteinase K (50 μg/mL final concentration at 37°C for 60 min) was followed by the incubation with PTA (final PTA concentration 0.3% at 37°C for 75 min). Samples were centrifuged and resultant pellets were resuspended in water and SDS-PAGE sample loading buffer. Approximately 27 - 50 mg of hemal nodes and 6 - 40 mg retropharyngeal lymph nodes (wet weight equivalent) were loaded onto each well of 12% Nu-PAGE Bis-Tris gels (Invitrogen, Carlsbad, CA). After electrophoresis, proteins were transferred onto PVDF membranes, blocked with commercial casein blocker (Pierce, Rockford, IL) and incubated with primary mAb F99/97.6.1 (3.5 μg/mL) followed by incubation with a horseradish peroxidase conjugated goat anti-mouse secondary Ab (1:5,000 dilution; SouthernBiotech, Birmingham, AL). Bound antibody was detected by chemiluminescence (Amersham ECL™, GE healthcare, Piscataway, NJ). Membranes were exposed to radiographic films (KodakBioMax Chemiluminescence Films) and evaluated for molecular masses of di-, mono- and un-glycosylated PrP^Sc^ bands using an Alpha Innotec image analyzer (Alpha Innotech Corp, San Leandro, CA). Hemal nodes and retropharyngeal lymph nodes collected from scrapie uninfected sheep were used as negative control for the western blot assay.

## Competing interests

All authors declared that they have no competing interests.

## Authors’ contributions

KO, RD and MO designed the experiments. KO, RD and DS analyzed the experimental data. RD prepared the manuscript. KO supervised the experiments and helped draft manuscript. TT prepared the tissues, developed sections for immunolabeling, performed all the immunohistochemistry assays and photomicroscopy. DZ performed western blot assays. MO helped draft manuscript. DS performed all the post mortems and helped draft the manuscript. All authors have read and approved the final manuscript.
